# *trans*-Carbonyl­chloridobis[tris­(4-chloro­phen­yl)phosphane]rhodium(I) acetone monosolvate

**DOI:** 10.1107/S1600536810039814

**Published:** 2010-10-09

**Authors:** Andrew R. Burgoyne, Reinout Meijboom, Alfred Muller, Bernard O. Omondi

**Affiliations:** a Research Centre for Synthesis and Catalysis, Department of Chemistry, University of Johannesburg, PO Box 524 Auckland Park, Johannesburg, 2006, South Africa

## Abstract

The title compound, *trans*-[RhCl(C_18_H_12_Cl_3_P)_2_(CO)]·C_3_H_6_O, contains an Rh(I) atom in a distorted square-planar coordination with a P—Rh—P angle of 175.27 (2)° and Rh—P bond lengths of 2.3127 (4) and 2.3219 (4) Å. The rhodium complexes link each other through weak inter­molecular contacts between the acetone methyl groups and the carbonyl O atom. Inter­actions between the acetone solvent mol­ecule and the Cl—Rh unit results in a reduced P—Rh—Cl angle of 86.675 (15)°.

## Related literature

For a review of rhodium Vaska {*trans*-[RhCl(CO)(P*R*_3_)_2_]} compounds, see: Roodt *et al.* (2003 ▶). For related compounds, see: Angoletta (1959 ▶); Vaska & Di Luzio (1961 ▶); Chen *et al.* (1991 ▶); Kuwabara & Bau (1994 ▶); Otto *et al.* (2000 ▶); Otto (2001 ▶); Meijboom *et al.* (2005 ▶).
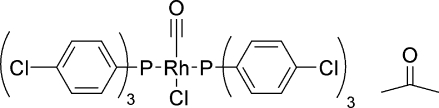


## Experimental

### 

#### Crystal data

[RhCl(C_18_H_12_Cl_3_P)_2_(CO)]·C_3_H_6_O
*M* *_r_* = 955.64
Triclinic, 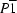

*a* = 10.6130 (7) Å
*b* = 12.7970 (8) Å
*c* = 16.7470 (11) Å
α = 71.631 (1)°
β = 81.742 (1)°
γ = 68.537 (1)°
*V* = 2007.9 (2) Å^3^
*Z* = 2
Mo *K*α radiation
μ = 1.01 mm^−1^
*T* = 100 K
0.37 × 0.17 × 0.07 mm

#### Data collection

Bruker X8 APEXII 4K KappaCCD diffractometer
Absorption correction: multi-scan (*SADABS*; Bruker, 2007 ▶) *T*_min_ = 0.707, *T*_max_ = 0.933
25954 measured reflections
10031 independent reflections
9160 reflections with *I* > 2σ(*I*)
*R*_int_ = 0.023

#### Refinement

*R*[*F* ^2^ > 2σ(*F* ^2^)] = 0.026
*wR*(*F* ^2^) = 0.069
*S* = 1.04
10031 reflections
471 parameters
4 restraints
H-atom parameters constrained
Δρ_max_ = 1.17 e Å^−3^
Δρ_min_ = −0.69 e Å^−3^

### 

Data collection: *APEX2* (Bruker, 2007 ▶); cell refinement: *SAINT-Plus* (Bruker, 2007 ▶); data reduction: *SAINT-Plus* and *XPREP* (Bruker, 2007 ▶); program(s) used to solve structure: *SHELXS97* (Sheldrick, 2008 ▶); program(s) used to refine structure: *SHELXL97* (Sheldrick, 2008 ▶); molecular graphics: *DIAMOND* (Brandenburg & Putz, 2005 ▶) and *ORTEP-3* (Farrugia, 1997 ▶); software used to prepare material for publication: *WinGX* (Farrugia, 1999 ▶).

## Supplementary Material

Crystal structure: contains datablocks global, I. DOI: 10.1107/S1600536810039814/si2294sup1.cif

Structure factors: contains datablocks I. DOI: 10.1107/S1600536810039814/si2294Isup2.hkl

Additional supplementary materials:  crystallographic information; 3D view; checkCIF report

## Figures and Tables

**Table:** 

C37—O1	1.1420 (18)
C37—Rh1	1.8177 (13)
P1—Rh1	2.3127 (4)
P2—Rh1	2.3219 (4)
Cl1—Rh1	2.3611 (4)

**Table:** 

O1—C37—Rh1	179.52 (16)
P1—Rh1—P2	175.268 (15)
C37—Rh1—Cl1	178.21 (5)
P1—Rh1—Cl1	86.675 (15)
P2—Rh1—Cl1	90.348 (15)

**Table 2:** Hydrogen-bond geometry (Å, °)

*D*—H⋯*A*	*D*—H	H⋯*A*	*D*⋯*A*	*D*—H⋯*A*
C15—H15⋯O2^i^	0.95	2.55	3.492 (3)	170
C21—H21⋯O2^ii^	0.95	2.34	3.267 (3)	165
C24—H24⋯Cl5^iii^	0.95	2.80	3.4734 (19)	129
C30—H30⋯Cl4^iv^	0.95	2.76	3.575 (2)	145
C35—H35⋯O1^v^	0.95	2.47	3.384 (2)	161
C40—H40*B*⋯Cl1	0.98	2.79	3.738 (3)	162

Symmetry codes: (i) 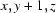; (ii) 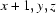; (iii) 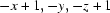; (iv) 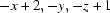; (v) 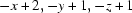.

**Fig. 1.**
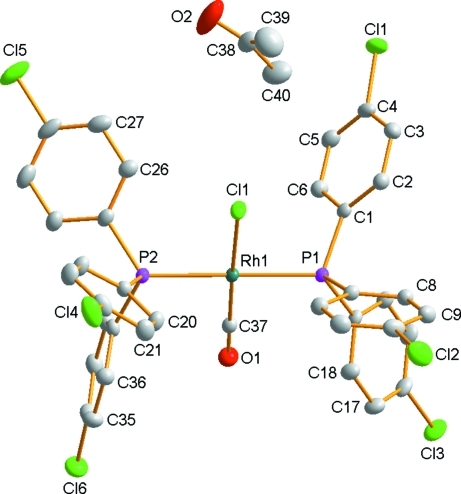
View of (I) (50% probability displacement ellipsoids) with H atoms omitted for clarity. The carbon atoms have been numbered sequentially and some labels have been omitted for clarity.

## supplementary crystallographic information

### Comment

The original Vaska complex, *trans*-[IrCl(CO)(PPh_3_)_2_], was first reported in 1959 (Angoletta, 1959), but later correctly formulated by Vaska in 1961 (Vaska & Di Luzio, 1961). This class of symmetrical square-planar complexes often crystallizes with the metal atom on a crystallographic centre of symmetry, thus imposing a disordered packing arrangement (Otto, 2001; Otto *et al.*, 2000; Chen *et al.*, 1991; Kuwabara & Bau, 1994). We report the title compound as one of the few crystallographic examples of this group which does not show disorder along the carbonyl/chloro axis.

Compound (I) crystallizes in a triclinic space group. The crystal structure of *trans*-[RhCl(CO){P(4-ClC_6_H_4_)_3_}_2_] (Fig.1) shows the expected square planar geometry with the phosphine ligands *trans* to each other.

As seen in Fig. 1 and Table 1, the complex shows a non-equally spaced square planar coordination centre with the P—Rh—P angle being 175.27 (2) °. The ^1^J(Rh—P) coupling of 128 Hz is in agreement with the coupling constants for other rhodium Vaska type complexes of this nature (Meijboom *et al.*, 2005).

Table 1 shows the angles C37—Rh—P1 and C37—Rh—P2 are nearly equal, while P2—Rh—Cl is 90.348 (15) ° and P1—Rh—Cl is only 86.675 (15) °. This inequality around the chlorine's displacement is interesting as the most linear hydrogen bond involving Cl1 is between C40—H40···Cl1 (162 °, Table 2) pulling it towards C40—H40 and thus decreasing the angle of P1—Rh—Cl. Additionally, the rhodium complexes link each other *via* weak intermolecular C—H···O and C—H···Cl hydrogen bonds (Table 2). The weak interactions between the acetone solvent molecule and the Rh—Cl unit resulted in a reduced P—Rh—Cl angle of 86.675 (15)°.

The ν(CO) stretching frequency of compound (1) is 1985 cm^-1^ which is higher than that of [Rh(CO)Cl{P(C_6_H_5_)_3_}_2_], the unchloronated version, which was reported by Roodt (Roodt *et al.*, 2003) as 1979 cm^-1^. The increase is due to the moderate electron withdrawing capabilities of the *para*-substituted chlorine on the phenyl rings.

### Experimental

To a solution of [Rh_2_(CO)_4_Cl_2_] (19.7 mg, 0.051 mmol) in acetone (3 cm^3^) was added 4 equivalents of a solution of P(4-ClC_6_H_4_)_3_ ligand (73.7 mg, 0.203 mmol) in acetone (2 cm^3^) while stirring. Immediately the effervescence of a gas, presumably CO, was observed. The solvent was evaporated in a round bottomed flask overnight yielding 62% of the title compound (28.2 mg, 0.0314 mmol). IR (cm^-1^): 629, 705, 746, 815, 1012, 1082, 1120, 1183, 1226, 1363, 1387, 1479, 1560, 1577, 1638, 1706, 1985 (CO). ^13^C NMR (δ /p.p.m., CDCl_3_): 139 (1 C, Ph), 137 (1 C, Ph), 135 (1 C, Ph), 133 (1 C, Ph), 130 (4 C, Ph), 129 (1 C, CO), 129 (1 C, Ph), 128 (1 C, Ph). ^31^P NMR (δ /p.p.m., CDCl_3_): 27.56 [^1^J(Rh—P) = 128 Hz].

### Refinement

The methyl and aromatic H atoms were placed in geometrically idealized positions and constrained to ride on their parent atoms, with C—H = 0.95–0.98 Å and *U*_iso_(H) = 1.5*U*_eq_(C) and 1.2*U*_eq_(C), respectively. The methyl groups were generated to fit the difference electron density and the groups were then refined as rigid rotors.

### Crystal data

**Table:**

[RhCl(C_18_H_12_Cl_3_P)_2_(CO)]·C_3_H_6_O	*Z* = 2
*M**_r_* = 955.64	*F*(000) = 960
Triclinic, *P*1	*D*_x_ = 1.581 Mg m^−^^3^
Hall symbol: -P 1	Mo *K*α radiation, λ = 0.71073 Å
*a* = 10.6130 (7) Å	Cell parameters from 25954 reflections
*b* = 12.7970 (8) Å	θ = 1.8–28.4°
*c* = 16.7470 (11) Å	µ = 1.01 mm^−^^1^
α = 71.631 (1)°	*T* = 100 K
β = 81.742 (1)°	Block, yellow
γ = 68.537 (1)°	0.37 × 0.17 × 0.07 mm
*V* = 2007.9 (2) Å^3^	

### Data collection

**Table:**

Bruker X8 APEXII 4K KappaCCD diffractometer	9160 reflections with *I* > 2σ(*I*)
graphite	*R*_int_ = 0.023
φ and ω scans	θ_max_ = 28.4°, θ_min_ = 1.8°
Absorption correction: multi-scan (*SADABS*; Bruker, 2007)	*h* = −14→12
*T*_min_ = 0.707, *T*_max_ = 0.933	*k* = −16→17
25954 measured reflections	*l* = −21→22
10031 independent reflections	

### Refinement

**Table:**

Refinement on *F*^2^	Primary atom site location: structure-invariant direct methods
Least-squares matrix: full	Secondary atom site location: difference Fourier map
*R*[*F*^2^ > 2σ(*F*^2^)] = 0.026	Hydrogen site location: inferred from neighbouring sites
*wR*(*F*^2^) = 0.069	H-atom parameters constrained
*S* = 1.04	*w* = 1/[σ^2^(*F*_o_^2^) + (0.0302*P*)^2^ + 1.7506*P*] where *P* = (*F*_o_^2^ + 2*F*_c_^2^)/3
10031 reflections	(Δ/σ)_max_ = 0.009
471 parameters	Δρ_max_ = 1.17 e Å^−^^3^
4 restraints	Δρ_min_ = −0.69 e Å^−^^3^

### Special details

**Table:**

Experimental. The intensity data was collected on a Bruker X8 Apex II CCD diffractometer using an exposure time of 10 s/frame. A total of 1895 frames were collected with a frame width of 0.5° covering up to θ = 28.38° with 99.9% completeness accomplished.
Geometry. All e.s.d.'s (except the e.s.d. in the dihedral angle between two l.s. planes) are estimated using the full covariance matrix. The cell e.s.d.'s are taken into account individually in the estimation of e.s.d.'s in distances, angles and torsion angles; correlations between e.s.d.'s in cell parameters are only used when they are defined by crystal symmetry. An approximate (isotropic) treatment of cell e.s.d.'s is used for estimating e.s.d.'s involving l.s. planes.
Refinement. Refinement of *F*^2^ against ALL reflections. The weighted *R*-factor *wR* and goodness of fit *S* are based on *F*^2^, conventional *R*-factors *R* are based on *F*, with *F* set to zero for negative *F*^2^. The threshold expression of *F*^2^ > σ(*F*^2^) is used only for calculating *R*-factors(gt) *etc*. and is not relevant to the choice of reflections for refinement. *R*-factors based on *F*^2^ are statistically about twice as large as those based on *F*, and *R*- factors based on ALL data will be even larger.

### Fractional atomic coordinates and isotropic or equivalent isotropic displacement parameters (Å^2^)

**Table:**

	*x*	*y*	*z*	*U*_iso_*/*U*_eq_	
C1	0.45279 (16)	0.41780 (13)	0.86206 (10)	0.0164 (3)	
C2	0.41530 (18)	0.37176 (15)	0.94513 (11)	0.0210 (3)	
H2	0.4811	0.3368	0.9874	0.025*	
C3	0.28161 (19)	0.37668 (16)	0.96678 (12)	0.0245 (4)	
H3	0.2559	0.3451	1.0234	0.029*	
C4	0.18729 (17)	0.42829 (16)	0.90434 (12)	0.0232 (3)	
C5	0.22084 (18)	0.47737 (16)	0.82210 (12)	0.0245 (4)	
H5	0.1539	0.5149	0.7805	0.029*	
C6	0.35413 (18)	0.47109 (15)	0.80104 (11)	0.0214 (3)	
H6	0.3787	0.5035	0.7443	0.026*	
C7	0.74075 (16)	0.32026 (14)	0.90731 (10)	0.0171 (3)	
C8	0.73223 (17)	0.35235 (15)	0.98114 (11)	0.0198 (3)	
H8	0.6603	0.4195	0.9892	0.024*	
C9	0.82786 (18)	0.28702 (17)	1.04254 (11)	0.0245 (4)	
H9	0.8209	0.3078	1.0931	0.029*	
C10	0.93422 (19)	0.19046 (17)	1.02877 (12)	0.0261 (4)	
C11	0.94494 (18)	0.15729 (15)	0.95637 (12)	0.0249 (4)	
H11	1.0181	0.091	0.9481	0.03*	
C12	0.84757 (17)	0.22195 (15)	0.89602 (11)	0.0212 (3)	
H12	0.8536	0.1991	0.8464	0.025*	
C13	0.62608 (16)	0.55598 (13)	0.81044 (10)	0.0161 (3)	
C14	0.51081 (17)	0.64893 (15)	0.82071 (11)	0.0205 (3)	
H14	0.4265	0.6367	0.8359	0.025*	
C15	0.51770 (19)	0.75957 (15)	0.80891 (12)	0.0244 (4)	
H15	0.4384	0.823	0.8147	0.029*	
C16	0.6411 (2)	0.77582 (15)	0.78876 (11)	0.0232 (3)	
C17	0.7573 (2)	0.68537 (16)	0.77805 (13)	0.0263 (4)	
H17	0.8416	0.698	0.7638	0.032*	
C18	0.74917 (18)	0.57584 (15)	0.78836 (12)	0.0230 (3)	
H18	0.8283	0.5137	0.7803	0.028*	
C19	0.86583 (16)	0.11855 (14)	0.62309 (10)	0.0173 (3)	
C20	0.96226 (18)	0.10485 (16)	0.67746 (11)	0.0232 (3)	
H20	0.9571	0.1691	0.6962	0.028*	
C21	1.06604 (19)	−0.00186 (17)	0.70460 (12)	0.0276 (4)	
H21	1.133	−0.0108	0.7406	0.033*	
C22	1.06923 (19)	−0.09470 (16)	0.67782 (11)	0.0256 (4)	
C23	0.9774 (2)	−0.08305 (16)	0.62225 (12)	0.0266 (4)	
H23	0.9838	−0.1473	0.6031	0.032*	
C24	0.87527 (19)	0.02477 (15)	0.59474 (12)	0.0236 (3)	
H24	0.8115	0.0343	0.5563	0.028*	
C25	0.59960 (17)	0.22421 (14)	0.55503 (11)	0.0196 (3)	
C26	0.49901 (18)	0.19286 (16)	0.60992 (12)	0.0242 (4)	
H26	0.4953	0.192	0.6671	0.029*	
C27	0.4036 (2)	0.16278 (17)	0.58096 (14)	0.0302 (4)	
H27	0.335	0.1412	0.6181	0.036*	
C28	0.4105 (2)	0.16491 (16)	0.49772 (14)	0.0304 (4)	
C29	0.5097 (2)	0.19354 (18)	0.44249 (13)	0.0312 (4)	
H29	0.5133	0.1929	0.3856	0.037*	
C30	0.60475 (19)	0.22352 (17)	0.47133 (12)	0.0259 (4)	
H30	0.6738	0.2437	0.4338	0.031*	
C31	0.79052 (17)	0.34837 (14)	0.50272 (10)	0.0183 (3)	
C32	0.69971 (18)	0.44776 (16)	0.45032 (11)	0.0223 (3)	
H32	0.6058	0.4585	0.4557	0.027*	
C33	0.74499 (19)	0.53101 (16)	0.39047 (11)	0.0248 (4)	
H33	0.6828	0.5983	0.3551	0.03*	
C34	0.8822 (2)	0.51408 (16)	0.38325 (11)	0.0246 (4)	
C35	0.97511 (19)	0.41477 (17)	0.43215 (12)	0.0261 (4)	
H35	1.0692	0.4032	0.425	0.031*	
C36	0.92848 (18)	0.33212 (15)	0.49190 (11)	0.0220 (3)	
H36	0.9915	0.2637	0.5258	0.026*	
C37	0.69720 (17)	0.47391 (12)	0.63949 (10)	0.0180 (3)	
P1	0.62373 (4)	0.40975 (3)	0.82264 (3)	0.01420 (8)	
P2	0.72683 (4)	0.25790 (4)	0.59485 (3)	0.01550 (8)	
Cl1	0.63216 (5)	0.17010 (4)	0.79542 (3)	0.02401 (9)	
Cl2	1.06102 (5)	0.11330 (5)	1.10165 (3)	0.04090 (13)	
Cl3	0.65127 (5)	0.91247 (4)	0.77695 (3)	0.03312 (11)	
Cl4	1.18984 (6)	−0.23221 (5)	0.71789 (3)	0.03995 (13)	
Cl5	0.28900 (6)	0.13135 (5)	0.46052 (4)	0.04566 (15)	
Cl6	0.93897 (6)	0.62136 (4)	0.31201 (3)	0.03453 (11)	
Cl7	0.02340 (5)	0.42625 (5)	0.92807 (4)	0.03486 (11)	
Rh1	0.668804 (12)	0.340979 (10)	0.705687 (7)	0.01450 (4)	
O1	0.71508 (14)	0.55764 (11)	0.59840 (8)	0.0272 (3)	
C38	0.3033 (2)	0.03136 (18)	0.88771 (14)	0.0311 (4)	
C39	0.3764 (3)	−0.0610 (2)	0.96104 (18)	0.0542 (7)	
H39A	0.3645	−0.1349	0.9651	0.081*	
H39B	0.34	−0.038	1.0126	0.081*	
H39C	0.473	−0.071	0.9539	0.081*	
C40	0.2919 (3)	0.1538 (2)	0.87945 (19)	0.0463 (6)	
H40A	0.2352	0.2075	0.8318	0.07*	
H40B	0.3823	0.1605	0.8699	0.07*	
H40C	0.2506	0.174	0.9312	0.07*	
O2	0.2549 (2)	0.0074 (2)	0.83829 (16)	0.0709 (7)	

### Atomic displacement parameters (Å^2^)

**Table:**

	*U*^11^	*U*^22^	*U*^33^	*U*^12^	*U*^13^	*U*^23^
C1	0.0168 (7)	0.0131 (7)	0.0212 (8)	−0.0044 (6)	−0.0005 (6)	−0.0083 (6)
C2	0.0219 (8)	0.0212 (8)	0.0215 (8)	−0.0077 (6)	−0.0018 (6)	−0.0073 (6)
C3	0.0255 (9)	0.0272 (9)	0.0248 (9)	−0.0129 (7)	0.0046 (7)	−0.0105 (7)
C4	0.0173 (8)	0.0227 (8)	0.0354 (10)	−0.0090 (6)	0.0045 (7)	−0.0159 (7)
C5	0.0202 (8)	0.0237 (8)	0.0309 (9)	−0.0053 (7)	−0.0050 (7)	−0.0104 (7)
C6	0.0213 (8)	0.0206 (8)	0.0222 (8)	−0.0066 (6)	−0.0021 (6)	−0.0058 (6)
C7	0.0165 (7)	0.0148 (7)	0.0194 (7)	−0.0044 (6)	−0.0028 (6)	−0.0044 (6)
C8	0.0182 (8)	0.0202 (8)	0.0219 (8)	−0.0061 (6)	−0.0002 (6)	−0.0079 (6)
C9	0.0226 (8)	0.0317 (9)	0.0203 (8)	−0.0103 (7)	−0.0015 (7)	−0.0070 (7)
C10	0.0211 (8)	0.0276 (9)	0.0237 (9)	−0.0070 (7)	−0.0065 (7)	0.0019 (7)
C11	0.0205 (8)	0.0184 (8)	0.0292 (9)	−0.0015 (6)	−0.0027 (7)	−0.0031 (7)
C12	0.0206 (8)	0.0173 (8)	0.0245 (8)	−0.0042 (6)	−0.0014 (6)	−0.0069 (6)
C13	0.0200 (8)	0.0129 (7)	0.0169 (7)	−0.0055 (6)	−0.0023 (6)	−0.0056 (6)
C14	0.0195 (8)	0.0169 (8)	0.0260 (8)	−0.0048 (6)	−0.0023 (6)	−0.0083 (6)
C15	0.0260 (9)	0.0147 (8)	0.0318 (9)	−0.0019 (6)	−0.0047 (7)	−0.0100 (7)
C16	0.0351 (10)	0.0133 (7)	0.0237 (8)	−0.0102 (7)	−0.0065 (7)	−0.0042 (6)
C17	0.0264 (9)	0.0222 (8)	0.0346 (10)	−0.0128 (7)	−0.0008 (7)	−0.0087 (7)
C18	0.0203 (8)	0.0176 (8)	0.0326 (9)	−0.0061 (6)	0.0000 (7)	−0.0099 (7)
C19	0.0166 (7)	0.0168 (7)	0.0177 (7)	−0.0042 (6)	−0.0005 (6)	−0.0058 (6)
C20	0.0241 (9)	0.0229 (8)	0.0233 (8)	−0.0051 (7)	−0.0047 (7)	−0.0093 (7)
C21	0.0220 (9)	0.0317 (10)	0.0247 (9)	−0.0018 (7)	−0.0065 (7)	−0.0083 (7)
C22	0.0230 (9)	0.0217 (8)	0.0202 (8)	0.0029 (7)	−0.0002 (7)	−0.0029 (7)
C23	0.0302 (10)	0.0183 (8)	0.0298 (9)	−0.0023 (7)	−0.0027 (7)	−0.0112 (7)
C24	0.0248 (9)	0.0198 (8)	0.0272 (9)	−0.0039 (7)	−0.0068 (7)	−0.0098 (7)
C25	0.0176 (8)	0.0165 (7)	0.0251 (8)	−0.0024 (6)	−0.0053 (6)	−0.0085 (6)
C26	0.0218 (8)	0.0206 (8)	0.0311 (9)	−0.0062 (7)	−0.0041 (7)	−0.0084 (7)
C27	0.0218 (9)	0.0230 (9)	0.0469 (12)	−0.0078 (7)	−0.0057 (8)	−0.0093 (8)
C28	0.0251 (9)	0.0180 (8)	0.0511 (12)	−0.0031 (7)	−0.0179 (8)	−0.0118 (8)
C29	0.0332 (10)	0.0286 (10)	0.0345 (10)	−0.0045 (8)	−0.0150 (8)	−0.0138 (8)
C30	0.0242 (9)	0.0276 (9)	0.0281 (9)	−0.0059 (7)	−0.0055 (7)	−0.0125 (7)
C31	0.0203 (8)	0.0183 (7)	0.0163 (7)	−0.0049 (6)	0.0003 (6)	−0.0071 (6)
C32	0.0195 (8)	0.0233 (8)	0.0207 (8)	−0.0032 (6)	0.0001 (6)	−0.0069 (7)
C33	0.0284 (9)	0.0199 (8)	0.0202 (8)	−0.0021 (7)	−0.0017 (7)	−0.0049 (7)
C34	0.0319 (10)	0.0213 (8)	0.0213 (8)	−0.0120 (7)	0.0031 (7)	−0.0054 (7)
C35	0.0226 (9)	0.0268 (9)	0.0292 (9)	−0.0111 (7)	0.0015 (7)	−0.0061 (7)
C36	0.0209 (8)	0.0203 (8)	0.0234 (8)	−0.0061 (6)	−0.0017 (6)	−0.0050 (7)
C37	0.0184 (7)	0.0166 (7)	0.0203 (7)	−0.0053 (6)	−0.0007 (6)	−0.0075 (6)
P1	0.01491 (18)	0.01144 (17)	0.01693 (18)	−0.00364 (14)	−0.00065 (14)	−0.00590 (14)
P2	0.01585 (19)	0.01487 (18)	0.01667 (19)	−0.00427 (15)	−0.00100 (14)	−0.00663 (15)
Cl1	0.0373 (2)	0.01797 (18)	0.02145 (19)	−0.01497 (17)	0.00302 (16)	−0.00717 (15)
Cl2	0.0270 (2)	0.0523 (3)	0.0291 (2)	−0.0029 (2)	−0.01223 (19)	−0.0002 (2)
Cl3	0.0448 (3)	0.0168 (2)	0.0426 (3)	−0.01495 (19)	−0.0064 (2)	−0.00755 (18)
Cl4	0.0390 (3)	0.0304 (2)	0.0270 (2)	0.0134 (2)	−0.0045 (2)	−0.00510 (19)
Cl5	0.0371 (3)	0.0340 (3)	0.0742 (4)	−0.0102 (2)	−0.0292 (3)	−0.0170 (3)
Cl6	0.0432 (3)	0.0276 (2)	0.0313 (2)	−0.0178 (2)	0.0031 (2)	−0.00148 (19)
Cl7	0.0213 (2)	0.0458 (3)	0.0463 (3)	−0.0170 (2)	0.00739 (19)	−0.0223 (2)
Rh1	0.01681 (7)	0.01199 (6)	0.01609 (6)	−0.00515 (4)	0.00030 (4)	−0.00597 (4)
O1	0.0338 (7)	0.0195 (6)	0.0280 (7)	−0.0111 (5)	−0.0011 (5)	−0.0040 (5)
C38	0.0233 (9)	0.0279 (10)	0.0451 (12)	−0.0039 (7)	−0.0021 (8)	−0.0200 (9)
C39	0.0591 (17)	0.0391 (13)	0.0492 (15)	−0.0084 (12)	−0.0019 (12)	−0.0020 (11)
C40	0.0412 (13)	0.0286 (11)	0.0677 (17)	−0.0111 (10)	−0.0042 (12)	−0.0116 (11)
O2	0.0511 (12)	0.0709 (14)	0.1097 (18)	0.0001 (10)	−0.0314 (12)	−0.0635 (14)

### Geometric parameters (Å, °)

**Table:**

C1—C2	1.392 (2)	C22—C23	1.380 (3)
C1—C6	1.397 (2)	C22—Cl4	1.7434 (18)
C1—P1	1.8165 (17)	C23—C24	1.393 (2)
C2—C3	1.396 (2)	C23—H23	0.95
C2—H2	0.95	C24—H24	0.95
C3—C4	1.383 (3)	C25—C26	1.395 (3)
C3—H3	0.95	C25—C30	1.398 (2)
C4—C5	1.377 (3)	C25—P2	1.8221 (17)
C4—Cl7	1.7356 (18)	C26—C27	1.397 (3)
C5—C6	1.387 (2)	C26—H26	0.95
C5—H5	0.95	C27—C28	1.378 (3)
C6—H6	0.95	C27—H27	0.95
C7—C12	1.396 (2)	C28—C29	1.373 (3)
C7—C8	1.402 (2)	C28—Cl5	1.7438 (19)
C7—P1	1.8183 (17)	C29—C30	1.392 (3)
C8—C9	1.387 (2)	C29—H29	0.95
C8—H8	0.95	C30—H30	0.95
C9—C10	1.392 (3)	C31—C36	1.395 (2)
C9—H9	0.95	C31—C32	1.400 (2)
C10—C11	1.383 (3)	C31—P2	1.8235 (17)
C10—Cl2	1.7366 (19)	C32—C33	1.391 (2)
C11—C12	1.386 (2)	C32—H32	0.95
C11—H11	0.95	C33—C34	1.385 (3)
C12—H12	0.95	C33—H33	0.95
C13—C14	1.394 (2)	C34—C35	1.384 (3)
C13—C18	1.397 (2)	C34—Cl6	1.7426 (18)
C13—P1	1.8265 (16)	C35—C36	1.392 (2)
C14—C15	1.394 (2)	C35—H35	0.95
C14—H14	0.95	C36—H36	0.95
C15—C16	1.378 (3)	C37—O1	1.1420 (18)
C15—H15	0.95	C37—Rh1	1.8177 (13)
C16—C17	1.384 (3)	P1—Rh1	2.3127 (4)
C16—Cl3	1.7387 (17)	P2—Rh1	2.3219 (4)
C17—C18	1.390 (2)	Cl1—Rh1	2.3611 (4)
C17—H17	0.95	C38—O2	1.200 (3)
C18—H18	0.95	C38—C39	1.481 (3)
C19—C24	1.391 (2)	C38—C40	1.490 (3)
C19—C20	1.394 (2)	C39—H39A	0.98
C19—P2	1.8231 (17)	C39—H39B	0.98
C20—C21	1.392 (3)	C39—H39C	0.98
C20—H20	0.95	C40—H40A	0.98
C21—C22	1.384 (3)	C40—H40B	0.98
C21—H21	0.95	C40—H40C	0.98
			
C2—C1—C6	119.00 (15)	C23—C24—H24	119.7
C2—C1—P1	125.78 (13)	C26—C25—C30	119.21 (17)
C6—C1—P1	115.16 (12)	C26—C25—P2	119.51 (14)
C1—C2—C3	120.45 (16)	C30—C25—P2	121.21 (14)
C1—C2—H2	119.8	C25—C26—C27	120.16 (18)
C3—C2—H2	119.8	C25—C26—H26	119.9
C4—C3—C2	118.86 (17)	C27—C26—H26	119.9
C4—C3—H3	120.6	C28—C27—C26	119.00 (19)
C2—C3—H3	120.6	C28—C27—H27	120.5
C5—C4—C3	121.87 (16)	C26—C27—H27	120.5
C5—C4—Cl7	118.27 (15)	C29—C28—C27	122.19 (18)
C3—C4—Cl7	119.80 (14)	C29—C28—Cl5	118.59 (17)
C4—C5—C6	118.82 (17)	C27—C28—Cl5	119.22 (17)
C4—C5—H5	120.6	C28—C29—C30	118.81 (19)
C6—C5—H5	120.6	C28—C29—H29	120.6
C5—C6—C1	120.96 (16)	C30—C29—H29	120.6
C5—C6—H6	119.5	C29—C30—C25	120.61 (19)
C1—C6—H6	119.5	C29—C30—H30	119.7
C12—C7—C8	119.10 (15)	C25—C30—H30	119.7
C12—C7—P1	119.17 (13)	C36—C31—C32	118.67 (16)
C8—C7—P1	121.57 (12)	C36—C31—P2	120.49 (13)
C9—C8—C7	120.63 (16)	C32—C31—P2	119.87 (13)
C9—C8—H8	119.7	C33—C32—C31	120.96 (17)
C7—C8—H8	119.7	C33—C32—H32	119.5
C8—C9—C10	118.82 (17)	C31—C32—H32	119.5
C8—C9—H9	120.6	C34—C33—C32	118.87 (17)
C10—C9—H9	120.6	C34—C33—H33	120.6
C11—C10—C9	121.59 (17)	C32—C33—H33	120.6
C11—C10—Cl2	118.84 (15)	C35—C34—C33	121.55 (16)
C9—C10—Cl2	119.51 (15)	C35—C34—Cl6	119.26 (15)
C10—C11—C12	119.16 (17)	C33—C34—Cl6	119.19 (14)
C10—C11—H11	120.4	C34—C35—C36	119.00 (17)
C12—C11—H11	120.4	C34—C35—H35	120.5
C11—C12—C7	120.69 (17)	C36—C35—H35	120.5
C11—C12—H12	119.7	C35—C36—C31	120.89 (17)
C7—C12—H12	119.7	C35—C36—H36	119.6
C14—C13—C18	118.59 (15)	C31—C36—H36	119.6
C14—C13—P1	123.20 (13)	O1—C37—Rh1	179.52 (16)
C18—C13—P1	118.21 (12)	C1—P1—C7	108.94 (8)
C13—C14—C15	120.87 (16)	C1—P1—C13	103.78 (7)
C13—C14—H14	119.6	C7—P1—C13	101.87 (7)
C15—C14—H14	119.6	C1—P1—Rh1	108.60 (5)
C16—C15—C14	119.16 (16)	C7—P1—Rh1	114.06 (5)
C16—C15—H15	120.4	C13—P1—Rh1	118.80 (5)
C14—C15—H15	120.4	C25—P2—C19	104.16 (8)
C15—C16—C17	121.33 (16)	C25—P2—C31	105.01 (8)
C15—C16—Cl3	119.40 (14)	C19—P2—C31	104.90 (7)
C17—C16—Cl3	119.27 (14)	C25—P2—Rh1	119.28 (6)
C16—C17—C18	119.17 (17)	C19—P2—Rh1	110.52 (5)
C16—C17—H17	120.4	C31—P2—Rh1	111.77 (5)
C18—C17—H17	120.4	C37—Rh1—P1	91.58 (5)
C17—C18—C13	120.86 (16)	C37—Rh1—P2	91.37 (5)
C17—C18—H18	119.6	P1—Rh1—P2	175.268 (15)
C13—C18—H18	119.6	C37—Rh1—Cl1	178.21 (5)
C24—C19—C20	119.35 (16)	P1—Rh1—Cl1	86.675 (15)
C24—C19—P2	122.03 (13)	P2—Rh1—Cl1	90.348 (15)
C20—C19—P2	118.57 (13)	O2—C38—C39	120.8 (2)
C21—C20—C19	120.77 (17)	O2—C38—C40	122.3 (2)
C21—C20—H20	119.6	C39—C38—C40	116.8 (2)
C19—C20—H20	119.6	C38—C39—H39A	109.5
C22—C21—C20	118.33 (17)	C38—C39—H39B	109.5
C22—C21—H21	120.8	H39A—C39—H39B	109.5
C20—C21—H21	120.8	C38—C39—H39C	109.5
C23—C22—C21	122.22 (17)	H39A—C39—H39C	109.5
C23—C22—Cl4	118.67 (15)	H39B—C39—H39C	109.5
C21—C22—Cl4	119.08 (15)	C38—C40—H40A	109.5
C22—C23—C24	118.69 (17)	C38—C40—H40B	109.5
C22—C23—H23	120.7	H40A—C40—H40B	109.5
C24—C23—H23	120.7	C38—C40—H40C	109.5
C19—C24—C23	120.54 (17)	H40A—C40—H40C	109.5
C19—C24—H24	119.7	H40B—C40—H40C	109.5
			
C6—C1—C2—C3	1.3 (2)	C32—C33—C34—Cl6	−176.89 (14)
P1—C1—C2—C3	−175.68 (13)	C33—C34—C35—C36	−2.2 (3)
C1—C2—C3—C4	−0.2 (3)	Cl6—C34—C35—C36	176.83 (15)
C2—C3—C4—C5	−1.7 (3)	C34—C35—C36—C31	0.0 (3)
C2—C3—C4—Cl7	175.40 (14)	C32—C31—C36—C35	2.1 (3)
C3—C4—C5—C6	2.3 (3)	P2—C31—C36—C35	−166.64 (15)
Cl7—C4—C5—C6	−174.84 (14)	C2—C1—P1—C7	5.09 (17)
C4—C5—C6—C1	−1.0 (3)	C6—C1—P1—C7	−172.00 (12)
C2—C1—C6—C5	−0.7 (3)	C2—C1—P1—C13	−102.85 (15)
P1—C1—C6—C5	176.61 (14)	C6—C1—P1—C13	80.06 (13)
C12—C7—C8—C9	−0.5 (2)	C2—C1—P1—Rh1	129.86 (14)
P1—C7—C8—C9	−175.72 (13)	C6—C1—P1—Rh1	−47.23 (13)
C7—C8—C9—C10	1.4 (3)	C12—C7—P1—C1	123.09 (14)
C8—C9—C10—C11	−1.2 (3)	C8—C7—P1—C1	−61.67 (15)
C8—C9—C10—Cl2	175.78 (14)	C12—C7—P1—C13	−127.67 (14)
C9—C10—C11—C12	0.1 (3)	C8—C7—P1—C13	47.57 (15)
Cl2—C10—C11—C12	−176.89 (14)	C12—C7—P1—Rh1	1.59 (15)
C10—C11—C12—C7	0.8 (3)	C8—C7—P1—Rh1	176.83 (12)
C8—C7—C12—C11	−0.6 (3)	C14—C13—P1—C1	−7.18 (16)
P1—C7—C12—C11	174.71 (14)	C18—C13—P1—C1	173.94 (14)
C18—C13—C14—C15	0.2 (3)	C14—C13—P1—C7	−120.32 (15)
P1—C13—C14—C15	−178.63 (14)	C18—C13—P1—C7	60.80 (15)
C13—C14—C15—C16	−1.5 (3)	C14—C13—P1—Rh1	113.46 (14)
C14—C15—C16—C17	1.6 (3)	C18—C13—P1—Rh1	−65.42 (15)
C14—C15—C16—Cl3	−178.16 (14)	C26—C25—P2—C19	92.47 (15)
C15—C16—C17—C18	−0.4 (3)	C30—C25—P2—C19	−84.32 (16)
Cl3—C16—C17—C18	179.36 (15)	C26—C25—P2—C31	−157.52 (14)
C16—C17—C18—C13	−0.9 (3)	C30—C25—P2—C31	25.69 (16)
C14—C13—C18—C17	1.0 (3)	C26—C25—P2—Rh1	−31.31 (16)
P1—C13—C18—C17	179.91 (15)	C30—C25—P2—Rh1	151.90 (13)
C24—C19—C20—C21	−1.1 (3)	C24—C19—P2—C25	15.80 (16)
P2—C19—C20—C21	176.43 (14)	C20—C19—P2—C25	−161.65 (14)
C19—C20—C21—C22	−1.5 (3)	C24—C19—P2—C31	−94.30 (15)
C20—C21—C22—C23	3.3 (3)	C20—C19—P2—C31	88.26 (15)
C20—C21—C22—Cl4	−174.74 (15)	C24—C19—P2—Rh1	145.08 (13)
C21—C22—C23—C24	−2.4 (3)	C20—C19—P2—Rh1	−32.37 (15)
Cl4—C22—C23—C24	175.62 (15)	C36—C31—P2—C25	−139.07 (14)
C20—C19—C24—C23	2.0 (3)	C32—C31—P2—C25	52.33 (16)
P2—C19—C24—C23	−175.44 (14)	C36—C31—P2—C19	−29.59 (16)
C22—C23—C24—C19	−0.3 (3)	C32—C31—P2—C19	161.81 (14)
C30—C25—C26—C27	−0.9 (3)	C36—C31—P2—Rh1	90.20 (14)
P2—C25—C26—C27	−177.74 (14)	C32—C31—P2—Rh1	−78.39 (15)
C25—C26—C27—C28	−0.1 (3)	C1—P1—Rh1—C37	119.85 (8)
C26—C27—C28—C29	1.2 (3)	C7—P1—Rh1—C37	−118.47 (8)
C26—C27—C28—Cl5	−178.26 (14)	C13—P1—Rh1—C37	1.69 (8)
C27—C28—C29—C30	−1.2 (3)	C1—P1—Rh1—Cl1	−60.55 (6)
Cl5—C28—C29—C30	178.27 (15)	C7—P1—Rh1—Cl1	61.14 (6)
C28—C29—C30—C25	0.1 (3)	C13—P1—Rh1—Cl1	−178.70 (6)
C26—C25—C30—C29	0.9 (3)	C25—P2—Rh1—C37	−116.11 (8)
P2—C25—C30—C29	177.70 (14)	C19—P2—Rh1—C37	123.26 (8)
C36—C31—C32—C33	−2.2 (3)	C31—P2—Rh1—C37	6.83 (8)
P2—C31—C32—C33	166.64 (14)	C25—P2—Rh1—Cl1	64.39 (7)
C31—C32—C33—C34	0.1 (3)	C19—P2—Rh1—Cl1	−56.23 (6)
C32—C33—C34—C35	2.1 (3)	C31—P2—Rh1—Cl1	−172.66 (6)

### Hydrogen-bond geometry (Å, °)

**Table:**

*D*—H···*A*	*D*—H	H···*A*	*D*···*A*	*D*—H···*A*
C15—H15···O2^i^	0.95	2.55	3.492 (3)	170
C21—H21···O2^ii^	0.95	2.34	3.267 (3)	165
C24—H24···Cl5^iii^	0.95	2.80	3.4734 (19)	129
C30—H30···Cl4^iv^	0.95	2.76	3.575 (2)	145
C35—H35···O1^v^	0.95	2.47	3.384 (2)	161
C40—H40B···Cl1	0.98	2.79	3.738 (3)	162

Symmetry codes: (i) *x*, *y*+1, *z*; (ii) *x*+1, *y*, *z*; (iii) −*x*+1, −*y*, −*z*+1; (iv) −*x*+2, −*y*, −*z*+1; (v) −*x*+2, −*y*+1, −*z*+1.

## References

[bb1] Angoletta, M. (1959). *Gazz. Chim. Ital.* **89**, 2359–2361.

[bb2] Brandenburg, K. & Putz, H. (2005). *DIAMOND* Crystal Impact GbR, Bonn, Germany.

[bb3] Bruker (2007). *APEX2*, *SAINT-Plus* and *SADABS* BrukerAXS Inc, Madison, Wisconsin, USA.

[bb4] Chen, Y.-J., Wang, J.-C. & Wang, Y. (1991). *Acta Cryst.* C**47**, 2441–2442.

[bb5] Farrugia, L. J. (1997). *J. Appl. Cryst.* **30**, 565.

[bb6] Farrugia, L. J. (1999). *J. Appl. Cryst.* **32**, 837–838.

[bb7] Kuwabara, E. & Bau, R. (1994). *Acta Cryst.* C**50**, 1409–1411.

[bb8] Meijboom, R., Muller, A. & Roodt, A. (2005). *Acta Cryst.* E**61**, m1283–m1285.

[bb9] Otto, S. (2001). *Acta Cryst.* C**57**, 793–795. 10.1107/s0108270101006035 11443242

[bb10] Otto, S., Roodt, A. & Smith, J. (2000). *Inorg. Chim. Acta*, **303**, 295–299.

[bb11] Roodt, A., Otto, S. & Steyl, G. (2003). *Coord. Chem. Rev.* **245**, 121–137.

[bb12] Sheldrick, G. M. (2008). *Acta Cryst.* A**64**, 112–122. 10.1107/S0108767307043930 18156677

[bb13] Vaska, L. & Di Luzio, J. W. (1961). *J. Am. Chem. Soc.* **83**, 2784–2785.

